# Chain Extension of Poly(Lactic Acid) (PLA)–Based Blends and Composites Containing Bran with Biobased Compounds for Controlling Their Processability and Recyclability

**DOI:** 10.3390/polym13183050

**Published:** 2021-09-09

**Authors:** Maria-Beatrice Coltelli, Alice Bertolini, Laura Aliotta, Vito Gigante, Alessandro Vannozzi, Andrea Lazzeri

**Affiliations:** 1 Department of Civil and Industrial Engineering, University of Pisa, 56122 Pisa, Italy; alicebertolini95@hotmail.it (A.B.); laura.aliotta@dici.unipi.it (L.A.); vito.gigante@dici.unipi.it (V.G.); andrea.lazzeri@unipi.it (A.L.); 2 National Interuniversity Consortium of Materials Science and Technology (INSTM), 50121 Florence, Italy; alessandrovannozzi91@hotmail.it

**Keywords:** poly(lactic acid), poly(butylene succinate), bran, chain extender, epoxidized soybean oil, succinic acid, malic acid

## Abstract

The present work focused on the research, design, and study of innovative chain extender systems of renewable origin for PLA–based biocomposites, reinforced with wheat bran as filler. The majority of employed chain extender compounds belongs to fossil world, affecting the biodegradability property which characterizes biopolymers. The aim of this work was thus to find promising biobased and sustainable alternatives to provide the same enhancements. According to this objective, epoxidized soybean oil (ESO) was chosen as principal component of the chain extender systems, together with a dicarboxylic acid, malic acid (MA), or succinic acid (SA). The reactivity of the modifier systems was previously studied through thermogravimetric analysis (TGA) and IR spectroscopy, to hypothesize the reaction mechanism in bran–filled blends. Hence, small–scale extrusion was carried out to investigate the effects of ESO/MA and ESO/SA on formulations of different composition (both pure PLA blends and composites). The variation of melt fluidity parameters was analyzed to define the optimized concentration of modifier systems. A comparison between the effects on blends of designed biobased systems and the action of fossil–based Joncryl was performed, to understand if the developed green solutions could represent competitive and efficient substitutes. The modified composites were characterized in terms of mechanical tests, degradation and thermal studies (TGA and DSC), and morphological analysis (SEM), to figure out their main features and to understand their potential in possible industrial applications.

## 1. Introduction

Over the last years, the field of classical petro–based polymers was enriched by the so called biopolymers and bioplastics, with the purpose of exploiting and marketing new kinds of materials more sustainable and friendly for the environment, simpler to be recycled or re–used, in the context of the circular economy, whose aim is the valorization of waste products as new raw sources and the consequent reduction of pollution [1,2]. Biopolymers are interesting for their biodegradability and ease of recyclability, which nowadays can be pivotal in packaging applications. Moreover, they are interesting for nontoxicity and biocompatibility, which make them suitable to be employed in the personal care and biomedical fields [3,4]. A particular class of biopolymers is represented by biocomposites, consisting of natural fibers reinforced biopolymeric matrix that represent an alternative to conventional materials that may be non–renewable, recalcitrant, or manufactured by polluting processes [3,5,6].

When matrix and fibers are both biodegradable, the corresponding final waste results to be fully green, following an entirely sustainable pathway of life cycle and reducing carbon footprint. In fact, carbon dioxide, which is released at the end of life of the material, during composting, is absorbed again by plants to perform the photosynthesis process [7,8]. Most natural additives improve the biodegradability of bio–based polymers such as poly(lactic acid) (PLA). Under controlled composting conditions, the biodegradation rate is increased by hydrophilic fillers, for example starch, bran, high amounts of chitosan or kenaf fibers [9,10,11].

One of the most available agricultural waste is represented by bran, obtained by the cereal agricultural stream. In particular, wheat bran is currently the most abundant bran waste. In fact, bran content of wheat is approximately 15% of the whole grain. Assuming that all wheat for human food consumption is milled, the wheat bran by–product stream would account for about 150 million tons per year [12]. Hence its valorization in composites can be particularly interesting. Nevertheless, bran is a very complex filler on a compositional point of view. The chemical composition of wheat bran predominantly includes non–starch polysaccharides (approximately 38%), starch (approximately 19%), protein (approximately 18%), and lignin (approximately 6%), with the non–starch polysaccharides being approximately 70% arabinoxylans, approximately 19% cellulose and approximately 6% β–(1,3)/β–(1,4)–glucan [13,14,15,16]. Despite of wheat bran was found as a promising reinforcing filler in natural rubber [17], in PLA biocomposites, rice bran was reported to induce polymer chain scission [18] because of the increased water uptake of composites. The interaction between matrix and bran can be improved by surface modification of the bran, for instance using biobased waxes [19].

In the case of biocomposites, the modification of the polymeric matrix is necessary to improve the interactions with fillers whose role is playing a reinforcing action. An example of compatibilizer is represented by polymeric coupling agents which improve the adhesion of inorganic fillers to the polymer matrix by physical interactions or chemical bonds [20,21,22]. Another possibility is the employment of a chain extender, which allows to obtain by reactive extrusion an extended polymeric network rich of crosslinking points where fillers and fibers, chemically linked to the matrix, could better hang to it. This pathway results very efficient for PLA–based blend [21,23,24].

However, biobased and biodegradable polymers require time for the degradation process in industrial composting plants under specific conditions, and their production is also linked to territorial availability [8,9,25]. The mechanical recycling of bioplastics would be thus important in the end of life phase. In fact, the recyclable items could be turned into raw materials that can then be used to make new products without needing to synthesize completely new resources. These products, obtained from secondary raw materials, in their end of life could then be treated by composting or anaerobic digestion, thus increasing the lifetime of the material before transforming it in basic chemicals [26]. For PLA–based bioplastics, incineration, composting, and anaerobic digestion processes seem to be clearly underperforming if compared with mechanical recycling methods, from an environmental point of view [10,27].

In studies regarding recyclability by mechanical recycling, widely applied in the packaging field, recovered products are collected, washed, and reprocessed, undergoing multiple extrusions, to assess the durability of the material by accelerated thermal aging: the major problem derived from this kind of recycling is the poor hydrolytic and thermal stability of PLA, subjected thus to chain scission, associated to decrease in terms of average molecular weight and mechanical properties [28]. Reprocessing through multiple successive injection or compression cycles in the presence of humidity, even in low concentration, causes chain scission, crystallization, and onset of cracks in the polymer. Thus, the embrittlement makes it not suitable for being used again for its initial scope [29]. The use of chain extenders enables the modulation melt viscosity and thermal stability, avoiding earlier degradation. Chain extension is a type of reaction to increase molecular weight during polymer processing of condensation polymers, promoting in the case of use of multifunctional reagents also chain branching, by means of a sort of post polymerization during melt compounding [21,30]. The substances which are employed are called chain extenders, having two (case of amines, anhydrides, epoxies and carboxylic acids, which provide linear polymers) or more functionalities [31,32]. Chain extension usually works by reacting end–groups with bi– or multifunctional reactive components. In the latter case, linear structures change their topology to long chain branched structures [21].

The higher the percentage of chain extender, the higher the value of molecular weight and broader will be the processing window of the bioplastic, which it is known to be very narrow. In fact, especially during processing at very high temperatures, biopolymers tend to degrade, and their molecular weight decreases fast, thereby the employment of a chain extender can overcome this problem [33,34].

In the case of biopolymer blends, chain extender action can be explained also in terms of in situ reactive compatibilization because they improve the compatibility of components of polymeric blends which, as already described [35], are often not miscible between each other. Both polymer species have reactive end groups, so that, through chain extender action, a graft copolymer between the two polymer chains is formed [35]. At the same time, the compatibilization effect enhances mechanical properties [22].

One of the most used fossil–based chain extender agents in polymers reactive blending is known with the trade name of Joncryl ADR (styrene–acrylate–glycidyl methacrylate copolymer, thus with multifunctional epoxy functionality), produced by BASF Company. In the case of biodegradable polyesters blends, its presence maximizes melt strength of polymer, acting also as a potential compatibilizer, increasing the adhesion between the filler and the predominant phase [36,37]. It can be used to compatibilize PLA/poly(butylene succinate–co–adipate) (PBSA) and PLA/poly(butylene adipate–co–terephthalate) (PBAT) blends, during various extrusion processes, like injection molding. Joncryl reacts with hydroxyl and/or carboxyl terminal groups of PLA and PBSA, working as a bridging element between the two polymers, improving interface properties [26,37].

In particular, in PLA/PBSA blends, Joncryl revealed to be very useful to control the fluidity and the processability of the melt. In fact, with the increase of PBSA content, the melt fluidity of blends increased, but the addition of the chain extender helped to re–establish the original situation, because of the increase in molecular weight consequent to the branching reactions [38]. Considering that Joncryl is not biobased or biodegradable, it might be important to design, define, and exploit as largely as possible chain extenders of biobased origin to grant a full circularity of the material [33,39,40], also avoiding the potential formation of microplastics after composting [41]. In fact, during composting tests on PLA blends containing fossil and not compostable polycarbonate (PC) [42] it was noticed that the final percentage of degradation was similar to the percentage of PLA in the blend. This means that PC remains persistently in the compost. In general, non–degradable additives represent a persistent fraction in compost [43].

Possible biobased alternatives could be epoxidized cardanol–based prepolymers, modified vegetable oils (like hydroxylated soybean oil), oil–based diisocyanates, green diols and acids (like furan oligomer (FO)), and by isosorbide, an ester of organic alcohols and nitric acid, often used in medical field as excipient in the treatment of cardiovascular diseases. For example, cardanol is an eco–friendly agro by–product of the cashew industry and can be used as a plasticizer for PVC and PLA or as co–reagent of epichlorohydrin to obtain biobased epoxy networks through curing reaction [34,44]. Thus, epoxidized vegetable oils constitute a good alternative because of their wide availability. However, their effectiveness is limited, because they consist of molecules having a few epoxide groups for each molecule, less efficient than commercial fossil alternatives in increasing the molecular weight of polyesters. In fact, they are mainly added in PLA as plasticizers [39].

In researches regarding innovative biobased thermosets, biobased diisocyanates and acids, such as tannic acid [45], were used to induce the crosslinking of the epoxidized vegetable oil (EVO) [46]. Alternatively, due to their hydrophobic nature, EVOs might be grafted on fibers surface (usually hydrophilic) to increase the interfacial adhesion with the polymeric matrix [47].

The combination of epoxidized oils and renewable acids was never considered in biopolyester blends and composites, with the exception of Liu et al. [48]. This paper reported the use of polyphenolic tannic acid crosslinked epoxidized soybean oil oligomers for strengthening and toughening bamboo fibers reinforced PLA biocomposites.

The objective of the present work is selecting an alternative chain–extension reaction occurring in the melt that can replace the use of fossil–based epoxy oligomers with natural and biobased counterparts, to formulate a fully sustainable polymeric material.

The selected reaction, never studied before for biopolyester chain extension, is the one between epoxidized soybean oil (ESO) and biobased dicarboxylic acids (DCA), in particular malic acid (MA) and succinic acid (SA).

This reaction will be studied in two different contests. The first system consists of blends of biopolyesters and, as reference, a PLA/PBSA 60/40 blend was selected since it showed properties similar to polyolefins and thus promising to replace them in many applications [38]; the second system consists of biocomposites of PLA–based blends, containing short fibers coming from agricultural waste. In this case, wheat bran was considered a representative example of short fibrous and complex polysaccharidic–based waste.

The reaction will be studied to be applied as a reactive extrusion process to provide an efficient polymeric network and to control the melt fluidity, the compatibility and stability of final blends and composites. The effect onto thermomechanical properties will be also investigated comparing chain extended biocomposites with those obtained by using Joncryl.

## 2. Materials and Methods

### 2.1. Materials

In this work the following polymeric granules and additives were used:

- Poly(lactic acid), trade name Luminy LX175, produced by Total Corbion. It is a highly viscous, amorphous, and transparent PLA that appears as white pellets and contains about 4% of D–lactic acid and a molecular weight of 163,000. This PLA, according to the producer’s data sheet has a density of 1.24 g/cm^3^ and a melt flow index (MFI) of 6 g/10 min (210 °C, 2.16 kg).
- Poly(butylene succinate–co–adipate) (PBSA), trade name BioPBS FD92PM, purchased from Mitsubishi Chemical Corporation (Tokyo, Japan). It is a copolymer of succinic acid, adipic acid and 1,4–butandiol with a melt flow index (MFI) of 4 g/10 min (190 °C, 2.16 kg) and a density of 1.24 g/cm^3^.
- Wheat bran available from WEAREBIO is a light brown powder with a content of protein (crude) of 13.84% (p/p), dietary fiber soluble of 0.93% (p/p), and dietary fiber insoluble of 19.70% (p/p) (CAS number: 130498–22–5; density: 0.51 g/cm^3^).
- Joncryl ADR 4468, indicated as JONCRYL, produced by BASF, is an epoxy oligomer. It is an oligomeric chain extender of fossil origin, carrying 20 average epoxy groups per macromolecule which react with chain ends of polycondensates (epoxy equivalent weight: 310 g/mol). Its molecular weight is 7250 g/mol, the density is 1.08 g/cm^3^, and it appears as solid flakes.
- Epoxidized soybean oil ESO, commercialized by Alcoplast (AP), is a light–yellow viscous liquid soluble in alcohols with an epoxide number of 4, a density 0.994 g/cm^3^ and a molar mass 950 g/mol.
- L–malic acid (MA), from renewable sources, provided by OENO S.r.l. group, was used. It is a dicarboxylic acid that appears as a solid white powder, odorless, with a molecular weight of 134.1 g/mol, a melting point of 131 °C, a decomposition point >225 °C and a solubility in ethanol of 45.5 g/100 g at 20 °C.
- Succinic acid (SA), purchased by Carlo Erba Reagents S.A.S. (Dast Group), is an organic compound belonging to the family of (di)carboxylic acids. It appears as a white and odorless powder having a density of 1.56 g/cm^3^, a melting point of 185 °C, a decomposition point of 235 °C and a molecular weight of 118.1 g/mol.

In this research work, the reference blend was a binary blend 60 wt %. PLA Luminy LX175 and 40 wt %. PBSA BioPBS–FD92PM used in previous works [38].

### 2.2. Methods

The blends were prepared by adding the modified bran to the 60/40 PLA/PBSA by using a micro–compounder Haake Minilab II (Thermo Scientific Haake GmbH, Karlsruhe, Germany), that provided also torque data. After the introduction of the material, the melt, pushed by the screws, runs through a closed circuit (with the valve closed) for 1 min, during which the torque is measured as a function of time. In the tests, the rotating speed was 110 rpm and the processing temperature was 190 °C. The final torque value represents the most significant value for the sample as the melt stabilizes. With the opening of the valve, the material was recovered and used in a Haake MiniJet Mini–Injection Molding System to prepare the specimens needed for the tensile tests. The cylinder temperature was 190 °C and the mold temperature was 45 °C. In the test, a pressure ranged from 350 bar to 600 bar (according to the kind of material) was used for 15 s and a post pressure of 200 bar for 5 s was needed to obtain the necessary filling of the mold.

The blends compositions are listed in Table 1. On the basis of a previous work [38] a PLA–PBSA matrix containing 60 wt % of PLA and 40 wt % of PBSA was chosen due to the good starting mechanical properties. The samples are named in a synthetic way using the letter *b* to indicate the blend PLA/PBSA 60/40, whereas the letter *c* indicates the composites with bran at 20% by weight. Then the additive ESO, MA, SA, and Joncryl are indicated in samples names. The last number the total percentage by weight of the modifier (0.5, 1, 2, or 5). > (or <) indicates an excess (or defect) of epoxydic groups of ESO with respect to carboxylic groups of MA.

Wheat bran fibers were added to decrease polymer final cost and ESO alone or in combination with malic acid or succinic acid in different ratio were used to prepare modified bran maintaining the same starting weight percentage of PLA/PBSA blend (80 wt %) in final extrusion. Bran weight percentage varied thus every time according to the quantity of modifier.

Plasticizer and/or acid–based modifier systems were obtained firstly by dissolution in beaker with 150 mL of ethanol as solvent, progressively adding bran powder. The sample were then left upon mechanical agitation by magnetic stirring for an entire night, until total evaporation of the solvent. Then it was placed for 24 h in oven (60 °C), to eliminate any residue of ethanol or humidity in the final samples. The solid product obtained was grinded to obtain a fine powder and then put again in oven (60 °C) for 24 h, to get ready for Minilab extrusion.

The investigation of flow behavior was carried out with a CEAST Melt Flow Tester M20 (Instron, Canton, MA, USA) equipped with an encoder. The ISO1133D custom TTT was followed. The sample was preheated without weight for 40 s at 190 °C, then a weight of 2.160 kg is released on the piston and after 5 s a blade cuts the strand starting the real test. Through the encoder, every 3 s, an MVR measurement is recorded and the MFR was determined weighing the material.

Tensile tests, performed on Haake Type III specimens (25 mm × 5 mm × 1.5 mm) obtained with the Haake MiniJet, were carried out by an MTS Criterion model 43universal tensile testing machine (MTS System Corporation, Eden Praire, MN, USA). The machine was equipped with a 10 kN load cell and interfaced with a MTS elite software. The initial grip separation was 25 mm, and the deformation rate was set at 10 mm/min.

Thermal properties were investigated by differential scanning calorimetric analysis (DSC) using a Q200 TA–DSC (TA Instruments, New Castle, UK). The samples were quickly cooled very fast from room temperature to −70 °C (equilibrate to −70 °C) and kept at this temperature for 1 min. Then the samples were heated at 10 °C/min to 190 °C and held for 5 min to remove the thermal history. Subsequently, the samples were cooled again at 10 °C/min to −50 °C and held at this temperature for 1 min. A second cooling scan from −70 °C to 190 °C, at 10 °C/min, was carried out to record the crystallization and melting behaviors. Melting temperature (*T_m_*) and the cold crystallization temperature (*T*_cc_) of the blends were recorded at the maximum of the melting peak and at the minimum of the cold crystallization peak respectively. As a consequence, the enthalpies of melting and of the cold crystallization were determined from the corresponding peak areas in the thermograms. DSC analysis was performed considering only the second heating scan to disregard the thermal history of the material. The percentage of crystallinity of PLA *X*_cc*,PLA*_ can be obtained through the relation

(1) $$X_{CC,PLA}=\frac{\Delta H_{m,PLA}-\Delta H_{cc,\;\;PLA}}{\Delta H^{0}{}_{m,PLA}\cdot X}$$

where Δ*H_m,PLA_* and Δ*H_cc,PLA_* are the melting enthalpy and the enthalpy of cold crystallization of PLA obtained in J/g, *X* is the weight fraction of PLA that crystallizes and $\Delta H^{0}{}_{m,PLA}$ is the melting enthalpy of the 100% crystalline PLA, equal to 93 J/g [20].

SEM analyses were carried out on samples previously cryo–fractured along the cross–section with liquid nitrogen, to cause fragile fracture, ensuring a smoother surface available for the study. The instrument was FEI Quanta 450 ESEM FEG scanning electron microscope (SEM) (Thermo Fisher Scientific, Waltham, MA, USA), which has a resolution power of 3.5 nm and possibility of magnification until 300,000×. Samples were not conductive and were coated with a thin metallic layer prior to microscopy to avoid charge build up.

Infrared spectra were recorded in the 550–4000 cm^−1^ range with a Nicolet 380 Thermo Corporation Fourier Transform Infrared (FTIR) Spectrometer (Thermo Fisher Scientific, Waltham, MA, USA) equipped with smart Itx ATR (Attenuated Total Reflection) accessory with a diamond plate, collecting 128 scans at 4 cm^−1^ resolutions. ONMIC software was used to modify the intensity of spectra and to compare different spectra profiles. To perform the reactivity study the starting DCA and ESO ratio corresponding to the stochiometric ratio between epoxide and carboxylic groups was selected. The reagents were deposited on a Petri plate made in Teflon. Then they were treated at 60 °C in oven (to simulate the treatment drying) or at 190 °C in a compression molding press (to simulate melt processing conditions).

Thermogravimetric analysis (TGA) was performed in nitrogen gas atmosphere by using a TA Q–500 (TA Instruments, Waters LLC, New Castle, DE, USA). The samples, in form of pellets or powder of about 10 mg, were heated at 10 °C/min from 30 °C to 800 °C in order to investigate degradation features.

## 3. Results

### 3.1. Reactivity Study

In the present study the ESO and DCA are added in the melt PLA based blends or composites with bran at 190 °C. Preliminary investigations regarding the stability and reactivity of the different reagents was thus carried out.

**Figure 1:**
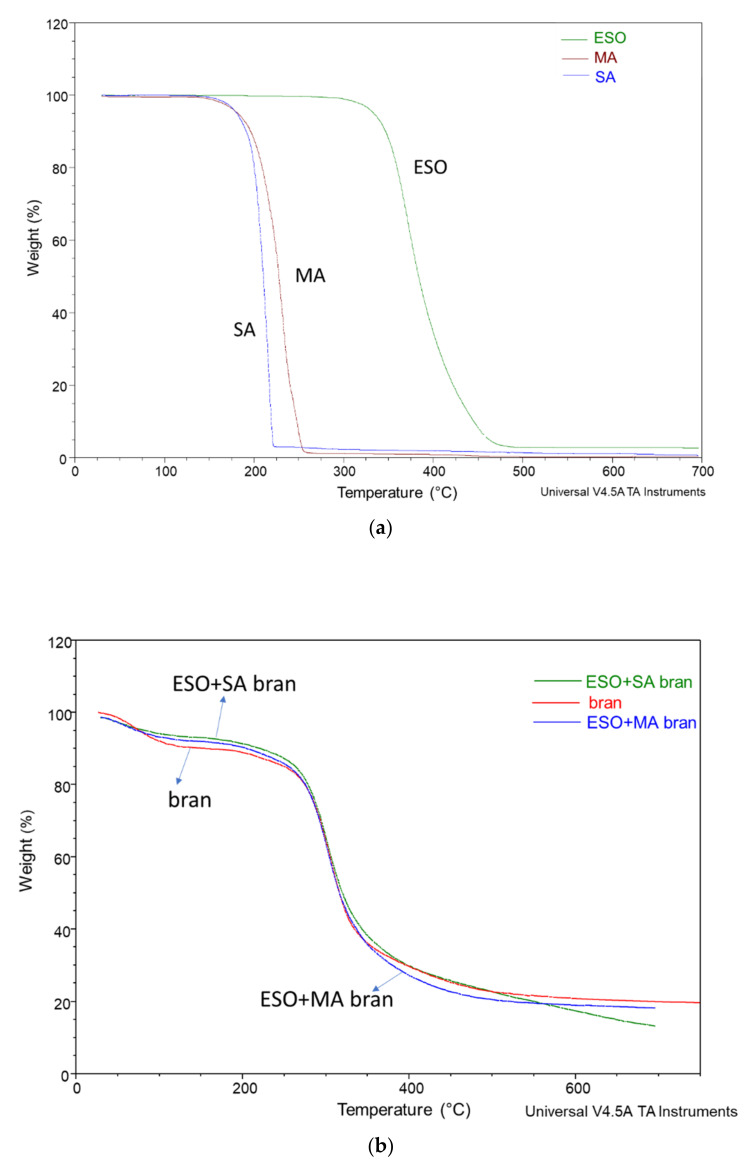
TGA curves related to (**a**) MA, SA and ESO; (**b**) bran, bran modified with ESO and MA and bran modified with ESO and SA.

The thermal stability in nitrogen atmosphere of the different reagents was studied by performing TGA measurements (Table 2). These investigations showed that ESO is thermally stable in the temperature range typical of PLA extrusion, as its onset temperature is 262.2 °C. Malic acid and succinic acid become unstable at 131 °C and 148 °C respectively, compatible with their respective melting points, but their peak temperature is well above 200 °C (Figure 1a). Hence some limited evaporation can be considered the reason of the observed slight weight loss and can be predicted also to occur during extrusion.

Wheat bran shows a first weight loss due to water. In the bran treated with ESO + MA or ESO + SA the water content decreased, probably because of the hydrophobic action of ESO that was deposited on the fibers. The onset and peak temperature were not significantly affected by the presence of DCA and ESO (Figure 1b).

**Figure 2:**
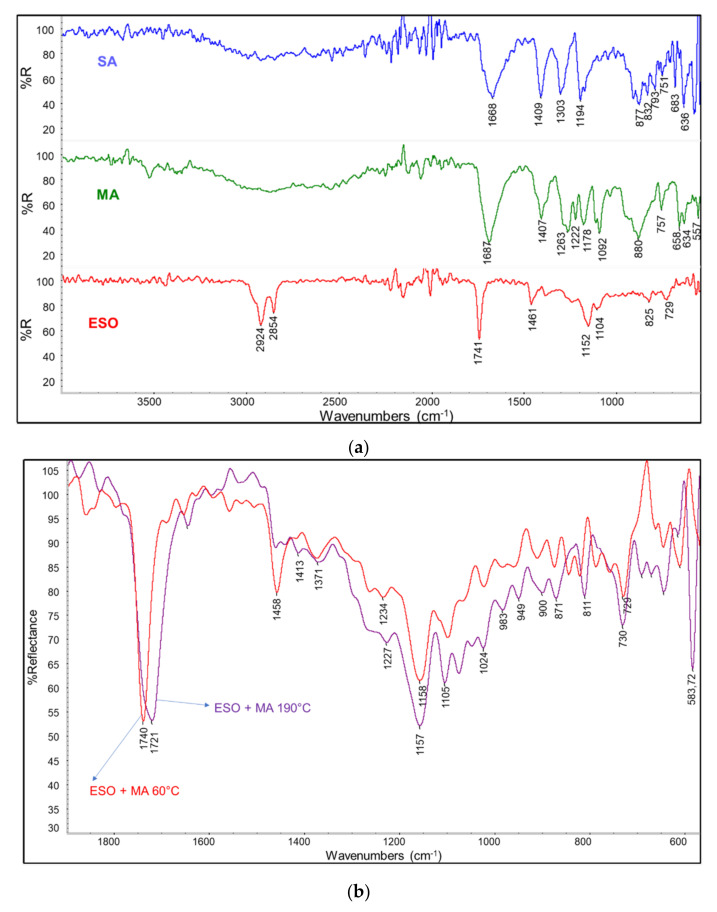

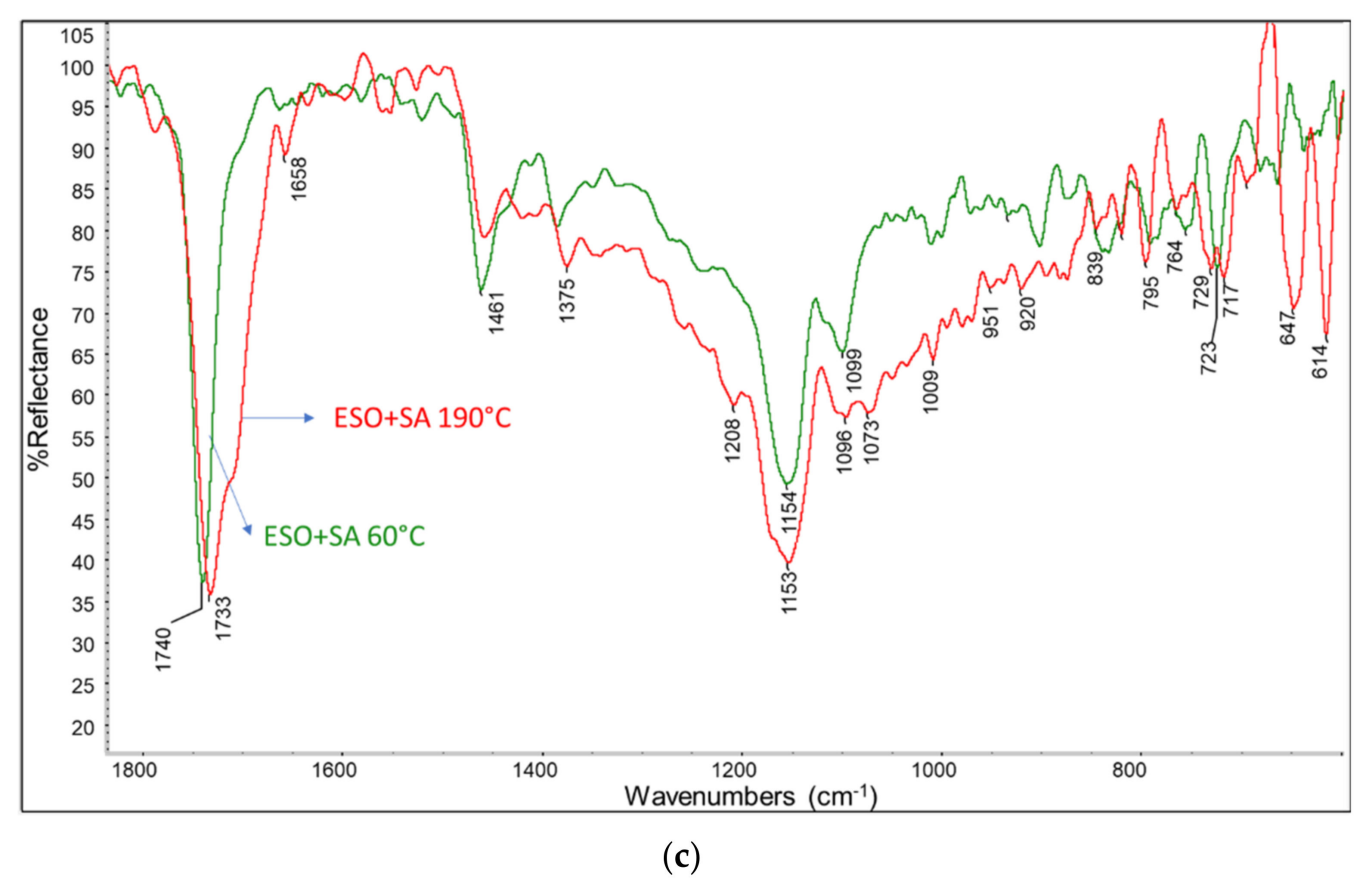
(**a**) infrared spectra of succinic acid (SA), malic acid (MA) and epoxidized soybean oil (ESO); (**b**) spectra of ESO and MA mixture recorded after thermal treatment at 60 °C and 190 °C; (**c**) spectra of ESO and SA mixture recorded after thermal treatment at 60 °C and 190 °C.

To observe how the ESO crosslinking in the presence of DCA undertook, some ATR IR spectra were collected, starting from the pure reagents (the malic acid/succinic solid powder and the liquid ESO, Figure 2a). In malic acid powder spectrum there is a broad central band in correspondence of 2873 cm^−1^, which is related to C–H stretching vibration. Moreover, at higher frequencies, the –OH stretching of hydroxyl and COOH groups can be observed. It is possible to observe the stretching vibration of –C=O (carbonyl bond) in correspondence of 1687 cm^−1^; a double peak, found at 1115 cm^−1^ (attributable to the stretching of –C–C=O bond) and at 1095 cm^−1^ (related to the stretching of the third isolated –C–O(H), instead at 930 cm^−1^ a strong diffuse band opens until 880 cm^−1^, indicating the out–of–plane deformation vibration of carboxylic acids. 

In the spectrum of SA powder, there is a broad band from about 3200 cm^−1^ to 2500 cm^−1^, which is typical of –OH stretching vibration for carboxylic acids; then a strongly intense peak can be observed in correspondence of 1669 cm^−1^, related to C=O symmetric stretching vibration, and three peaks related to anti–symmetric C–O stretching vibrations (1409, 1303, 1195 cm^−1^) in crystals, overlapped to COO symmetric vibration, and another diffuse band between 910 cm^−1^ and 790 cm^−1^, which can be associated to out–of–plane deformation vibration of the –OH in the carboxylic groups [49]. It is important to underline the characteristic presence of three strong subsequent peaks (at 683, 636, and 585 cm^−1^), representing the in–plane deformation of –O–C=O bonds in succinic acid skeleton [50].

In ESO spectrum there are two evident bands related to asymmetric and symmetric stretching of –CH_2_ (respectively at 2920 cm^−1^ and at 2855 cm^−1^). Other significative peaks are at 1740 cm^−1^ (stretching vibration of C=O, typical of triglycerides); 1461 cm^−1^ (bending of –CH_2_ inside chains); 1243 cm^−1^ (medium intensity, stretching vibration of –C–O bonds in epoxy rings); 1151 cm^−1^ (asymmetric stretching vibration of ester bonds C–O); then two small peaks, 834 cm^−1^ and 826. cm^−1^, belonging to the region characteristic of epoxy rings, indicate their presence along the chain. These peaks (Figure 2a) were summarized in Table 3.

The reaction between ESO and DCA in stoichiometric ratio was investigated on a Teflon layer placed upon a Petri plate by dissolving the reagents in ethanol and evaporating the solvent at 60 °C. The reaction had been simulated following the conditions used to superficially modify bran in the case of cESOMA1 and cESOSA1 formulations, respectively for MA and SA; the mixture was subjected to temperature increase, staying every time one hour at 60 °C, after one hour at 80 °C and at the end at 190 °C for 1 min (the extrusion temperature and duration). In particular, esterification occurred passing from 80 °C to 190 °C for both ESO/MA and ESO/SA systems. It was noticed that after the removal of ethanol a partially inhomogeneous material was obtained, reasonably because of DCA segregation in crystals inside the hydrophobic ESO. Hence the reaction occurred mainly above the melting temperature of DCA (131 °C for MA and 185 °C for SA), when the interactions between the ESO and DCA are maximined in a homogenous phase. A shrink and shift of –C=O stretching band from 1740 of ESO cm^−1^ to 1733–1720 cm^−1^ was observed (Figure 2b,c). This change can be attributed at the formation of linkages between the carboxylic groups of DCA and the ESO epoxide groups.

Looking at the spectrum obtained at 60 °C of ESO and MA (Figure 2b), it can be seen that the spectrum—apart from some peaks shifts due to reciprocal interaction between the two reagents—is reasonably the sum of the ESO spectrum with some minor bands attributable to MA, being the ESO the main component because of the selected stoichiometric ratio. The main C=O stretching peak is thus the one of ESO at 1740 cm^−1^. After the thermal treatment at 190 °C the main peak resulted shifted at 1720 cm^−1^. Moreover, the appearance of new bands in the 1000–1300 region can suggest the presence of different C–O stretching bands due to the formation of ester linkages between MA and ESO. The broad band is due to the formation of different ester bonds thanks to the reaction with epoxide groups of ESO that can involve carboxylic or hydroxyl groups of malic acid. However, the spectrum obtained after the treatment at 190 °C contains new bands that can induce at hypothesizing a complex mechanism, considering also the dehydration of MA to fumaric acid, reported by several authors [51,52], and occurring above 235 °C, in agreement with the appearance of the 1644 cm^−1^ band, attributable to C=C stretching and the characteristic intense band at 583 cm^−1^ reasonably attributable to skeletal torsional vibration of cis–alkenes [53].

Regarding the reaction occurring between ESO and SA, a similar shift of the C=O stretching band was observed (Figure 2c). In this case the new band is centered at 1733 cm^−1^ but a shoulder at lower wavenumber is also present. Moreover, new bands in the region 1000–1300 cm^−1^ of modest intensity were observed, in agreement with the formation of different ester groups. The other bands can be attributed to the ones of succinic acid that result shifted at lower wavenumbers due to the interactions occurring with ESO. The absence of a hydroxyl group with respect to MA would make the SA not subjected to dehydration. Anyway, succinic acid is reported to partially convert to succinic anhydride above its melting temperature [54]. However, the occurring of this reaction cannot be easily demonstrated by the ATR spectrum.

**Figure 3:**
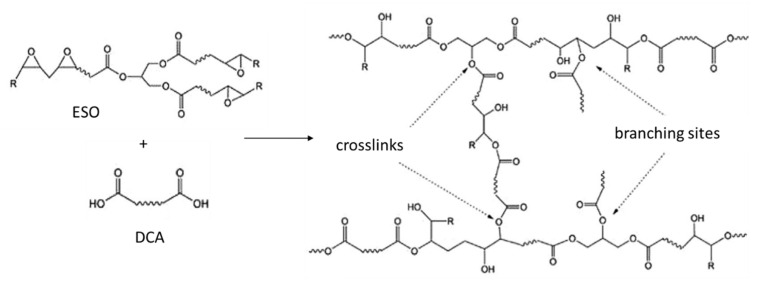
Scheme regarding the reaction between an epoxidized oil (EO) and DCA, resulting in cross–linking.

In general, the infrared characterization evidenced the esterification reaction occurring between the ESO and the dicarboxylic acids upon heating at 190 °C. The reaction is mainly attributable to the high reactivity of carboxylic groups with epoxide groups. However, hydroxyl groups can also react with epoxide groups. Hence, this reaction induces the formation of branched macromolecules. The opening of an epoxide group leads to formation of a free hydroxyl group, that can result in branching (Figure 3). Additionally, MA has an hydroxyl group. Hence, it can result certainly highly reactive towards ESO.

### 3.2. Melt Flow Study

The Torque and Melt Flow Rate (Table 4) of the different blends and composites was determined to investigate the effect of the additives on melt viscosity and fluidity, respectively.

**Figure 4:**
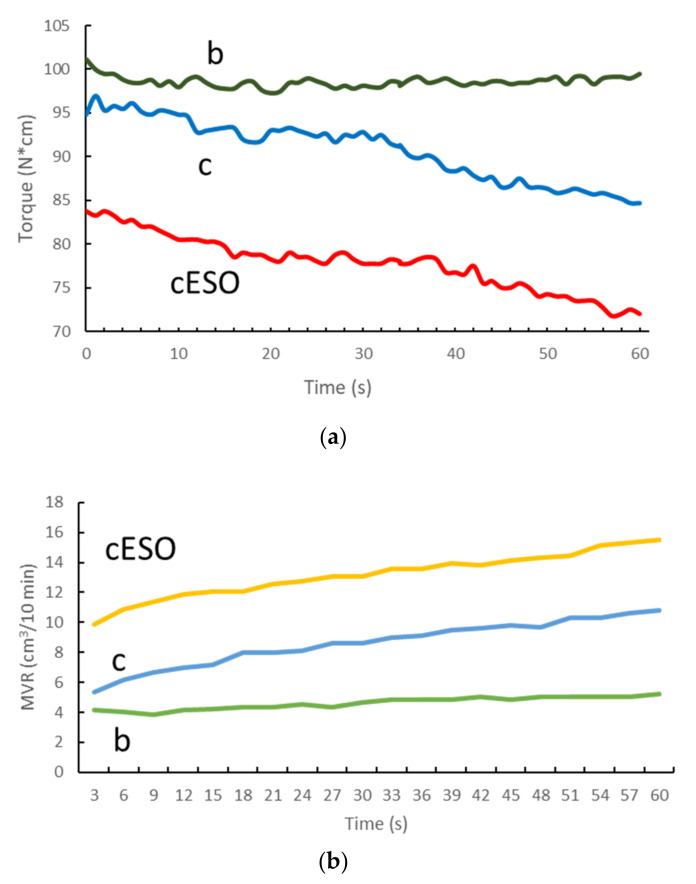
(**a**) Torque during extrusion and (**b**) melt volume rate trend as a function of testing time for the PLA/PBSA blend (b trend), the composite with 20% bran (c trend) and the latter with ESO (cESO trend).

By comparing the c composite containing bran with the b blend, it was evident a decrease in torque and increase in MVR during the extrusion and the Melt Flow Rate tests respectively (Figure 4), reasonably attributable at the occurrence of polyester chain scission due to hydroxyl surficial groups in bran or at the presence of residual humidity in bran, despite of the accurate drying before processing. The addition of ESO resulted in a decrease in Torque and in an increase in MFR, reasonably attributable to the plasticizing effect of the liquid epoxidized oil. In general, in the c composite the MVR recorded during the testing time shows an increasing trend more relevantly than b blend, indicating a lower stability of the material in the molten state. This instability can be attributed at the occurring of chain scission promoted by the bran nucleophilic groups also during the testing in the MFR instrument. In the absence of bran, the MVR is almost constant as a function of time.

**Figure 5:**
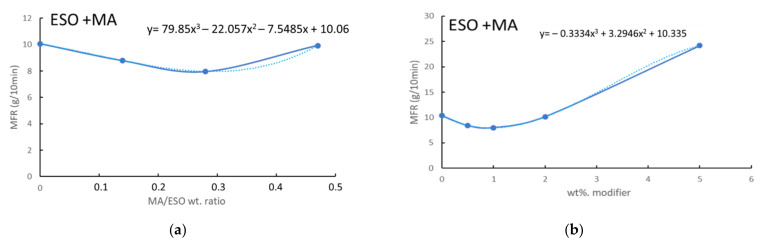

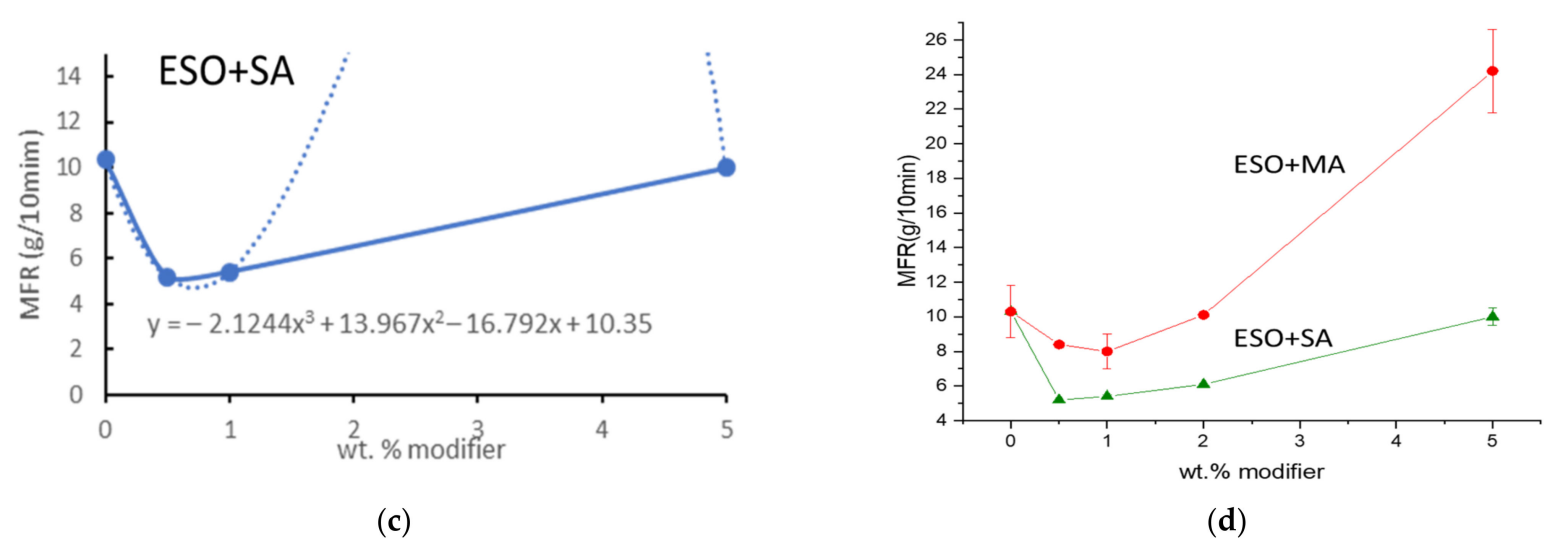
MFR data related to: (**a**) composites with ESO + MA as a function of MA/ESO weight ratio; (**b**) composites with ESO + MA as a function of modifier (ESO + MA, stoichiometric ratio) weight ratio; (**c**) composites with ESO + SA as a function of modifier (ESO + SA, stoichiometric ratio) weight ratio; (**d**) superposition of the trends reported in (**b**,**c**).

Initially, the different effects of stoichiometric ratio between ESO and MA (cESOMA1), ESO excess (cESO > MA1), and MA excess (cESO < MA1) were investigated by plotting the MFR as a function of the MA/ESO weight ratio (Figure 5a). The trend was fitted and the obtained equation showed a minimum at weight ratio MA/ESO of 0.29, very close to the stoichiometric ratio (0.28). Hence, the reactivity is maximized when the ratio between the –COOH groups of MA and the epoxidic groups of ESO is close to 1.

The MFR was studied as a function of the total content of modifier, consisting of both MA and ESO (Figure 5b). A fitting equation was obtained that showed a minimum value. The minimum trend can be justified considering that, by increasing the amount of DCA, the autocatalytic chain scission is more effective because of the increase in acidic groups concentration. The minimum value of MFR corresponded to a modifier content of 0.73%.

A similar plot was also considered in the case of ESO and SA (Figure 5c). The fitting resulted in an equation that was minimized when the total content of modifier was 0.72%. The superposition of the trends corresponding at the two DCA (Figure 5d) evidenced that the system MA + ESO is less efficient in decreasing the MFR than the SA + ESO system. This substantial difference is ascribable at the presence in MA of the hydroxyl group, that induces chain scission in the polyesters because of its nucleophilic character. Thus, on one hand, the coupling reaction between ESO and DCA induces a decrease in fluidity; but on the other hand, the autocatalytic effect of –COOH groups (as well as the nucleophilic behavior of the –OH group on MA) favors the chain scission during the processing.

**Figure 6:**
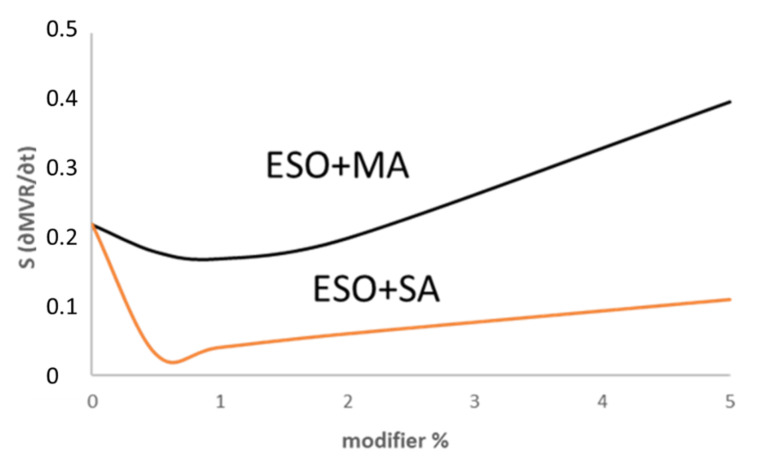
Slope of MVR (time) curves as a function of modifier wt % for bran composites treated with ESO + MA or ESO + SA.

MVR curves were recorded during the test for 1 min for each tested blend and composite (Figure 4b). The slope of the MVR trend can be considered an indication of the stability of the material in the melt (in view of further processing or recycling of the material). In fact, if any chain scission is occurring the MVR remains stable, whereas, if chain scission occurs, the MVR increases, hence its slope increases too. The slope of the MVR curves was plotted as a function of modifier weight percentage for the composites treated with ESO and MA and for the composites treated with ESO and SA (Figure 6). In the former case, a beneficial effect on stability is observed only in a limited range of concentration, reasonably because of the chain scission action of the hydroxyl group on the MA molecules. In the case of SA, the slope values are much lower, indicating a higher stability in the melt of the composites treated with ESO +SA. The values observed for the system consisting of ESO and SA are significantly lower than the ones observed for the composites with only ESO (intercept value). Hence the addition of SA, is beneficial for stability in all the explored concentration range, up to 5%.

**Figure 7:**
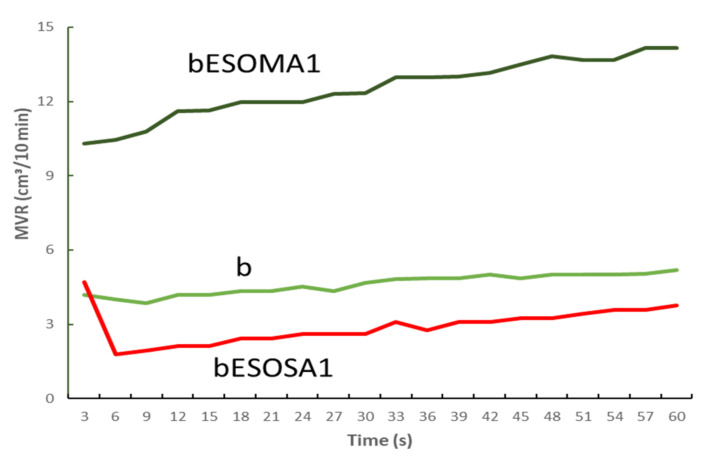
MVR trend of the PLA/PBSA 60/40 blend (b), the b blend with ESO + MA (bESOMA1) and the b blend with ESO + SA (bESOSA1).

The chain extension system consisting of ESO and SA was demonstrated to work well also in the PLA/PBSA blend, without bran (Figure 7). The MVR is reduced with respect to the value recorded for the blend. On the contrary, the extension system consisting of ESO and MA results mainly in chain scission, in agreement with the chain scission induced by the –OH group on MA.

**Figure 8:**
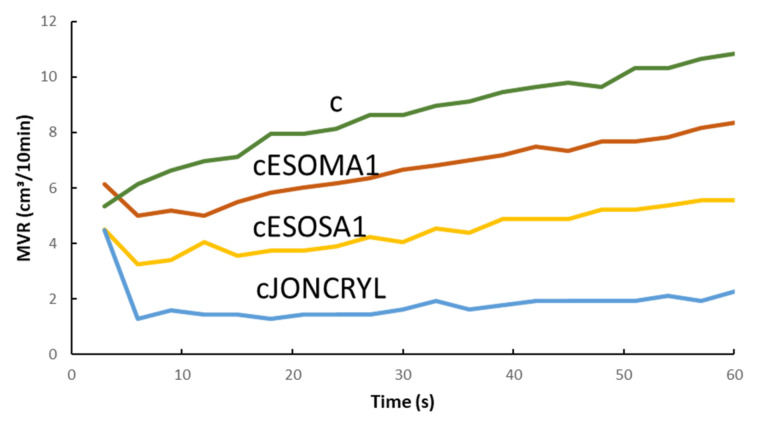
Comparison of MVR trends between the bran composite (c) and the composite chain extended with ESO + MA (cESOMA1), ESO + SA (cESOSA1) or Joncryl (cJONCRYL).

To conclude the melt fluidity analysis, a final PLA/PBSA blend was formulated adding bran fibers, but using as chain extender the common petro–based Joncryl, widely employed to enhance melt viscosity and stability of PLA–based blends, as already described. The purpose was comparing the bio–based chain extender systems ESO + MA and ESO + SA, to an efficient one of fossil origin (and thus not biodegradable). For reasons of continuity cESOMA1 and cESOSA1 were preferred to be considered, because both prepared with 1wt % of modifier system, like the formulation where Joncryl played the same role. Due to its high reactivity, Joncryl ensured better melt parameters (Figure 8) but ESO + SA–based formulation resulted quite reliable and competitive, especially looking at MVR value which oscillated between 4.5 and 5.5 cm^3^/10 min.

### 3.3. Thermal and Mechanical Characterization of Selected Composites

The effect of the chain extension on composites properties was investigated by the characterization of formulations which resulted to be the most significant, to outline their mechanical behavior (through tensile tests), thermal properties (by DSC analysis), thermal stability (by TGA analysis), and the morphology (by SEM analysis).

#### 3.3.1. Mechanical Tests

Tensile results (Table 5) showed that the addition of bran (sample c) induced a decrease above all in elongation at break with respect to the matrix blend (sample b). On the other hand, the tensile strength remains almost unchanged. The bran addition seems to act as a stress intensifier making the material more susceptible to fracture at low strains.

**Figure 9:**
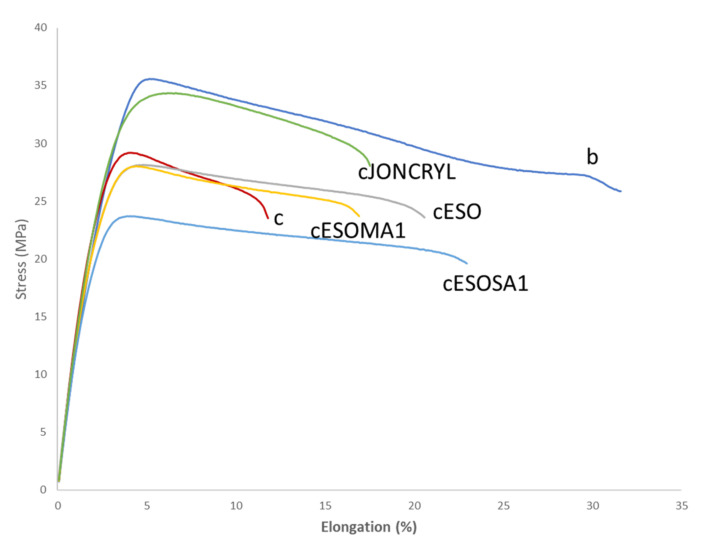
Representative stress–strain trends of selected formulations. b is the PLA/PBSA 60/40 blend; c is the composite with 20% bran; cESO is the composite plasticized with ESO; cESOMA1 is the composite with ESO + MA; cESOSA1 is the composite with ESO + SA; cJONCRYL is the composite with Joncryl.

With respect to c, the plasticizing action due to epoxidized oil (cESO) led to a ductility increase (Figure 9), whereas the elongation at break slightly decreased when ESO–MA was employed. ESO–SA presence ensured great elongation but slightly reduced tensile strength. Substantially, the properties were not significantly modified and can be considered intermediate between those of c and cESO. In fact, ESO is a liquid and can induce a plasticizing action. On the other hand, the formation of linkages, enabled by SA and MA and evidenced thanks to the melt fluidity analysis, counterbalanced the plasticizing effect of ESO. It is evident that Joncryl, in agreement with its higher reactivity, induced an increase in tensile strength with respect to c composite.

#### 3.3.2. DSC Analysis

The results of DSC tests (Table 6), related to the second heating scan, showed that the T_g_ of PLA in the b reference matrix attested at ~58 °C and slightly decreased to 55–56 °C in presence of bran fibers and other additives. In particular, this can be related to the plasticization effect of ESO and low molecular weight components of bran (it was absent in cJONCRYL, where ESO is not present, where T_g_ slightly increased with respect to c composite thanks to chain extension).

**Figure 10:**
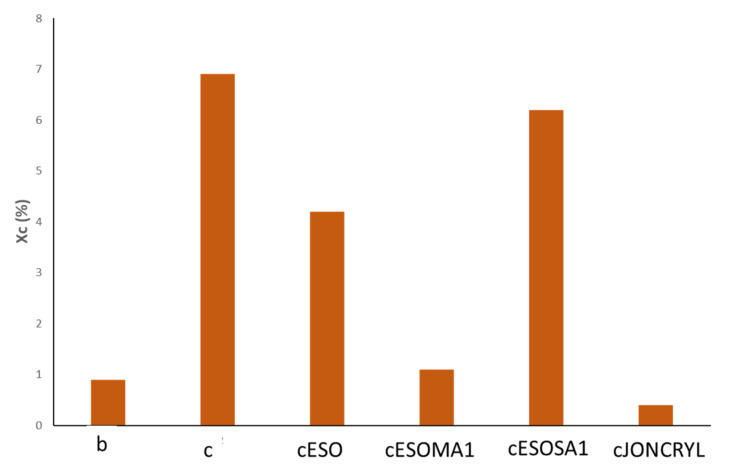
Crystallinity of the selected formulation (second heating scan). b is the PLA/PBSA 60/40 blend; c is the composite with 20% bran; cESO is the composite plasticized with ESO; cESOMA1 is the composite with ESO + MA; cESOSA1 is the composite with ESO + SA; cJONCRYL is the composite with Joncryl.

The c composite showed (Figure 10) the highest X_c_ value (6.9, always poor) suggesting that bran fibers, being natural fibers, could act as a nucleating agent. This effect is known in literature because they act as sites of heterogeneous nucleation [55,56,57]. This result was reduced in cESO due to ESO plasticizing action which increases the free volume between the amorphous part and the crystals [58]. Crystallization occurred also in cESOSA1, where succinic acid was present, instead in the case of malic acid (cESOMA1) it can be hypothesized that the occurred chain scission and presence of branched structures prevailed, introducing more disorder and thus hindering any possible ordered packing mechanism, thus increasing the amorphous character of the overall blend (X_c_ = 1.1). In cJONCRYL, probably Joncryl both reacted with biopolyesters and bran fibers, generating a general increase in structural disorder in the polymeric chains and also discouraging the matrix nucleation (X_c_ = 0.4).

#### 3.3.3. Thermogravimetric Analysis

Thermogravimetric investigations were carried out on the selected blends as well as on the pure PLA and PBSA polymers (Table 7).

Thermogravimetric analysis allowed to define thermal stability of the formulations, following the procedure described in 2.2. Usually, at the beginning of the test, volatile substances (humidity, solvents, or unreacted monomers) are lost, followed by the mass decomposition of the polymer itself, whereas ashes, inorganic compounds, fillers and/or fibers, which were not oxidized, constitute the inert residue. In fact, except for pure matrix b, it was possible to notice that TGA thermograms of other added blends were characterized by an initial drop related to the evaporation of water from bran filler surface.

Besides, with respect to b (T_on_ of about 261 °C), it was observed that degradation started earlier when bran fibers were added (c). ESO presence slightly increased T_on_ up to 247.6 °C (from 242.9 °C of c) while, in the cases of cESOMA1 and cESOSA1, where acids where used, onset temperatures decreased and were essentially comparable, even if succinic acid presence (cESOSA1) confirmed to be more reliable and helpful than malic acid (cESOMA1) in degradation terms, looking at weight loss and residue values; anyway, Joncryl action was the strongest in retarding degradation, ensuring a T_on_ value of 253 °C.

**Figure 11:**
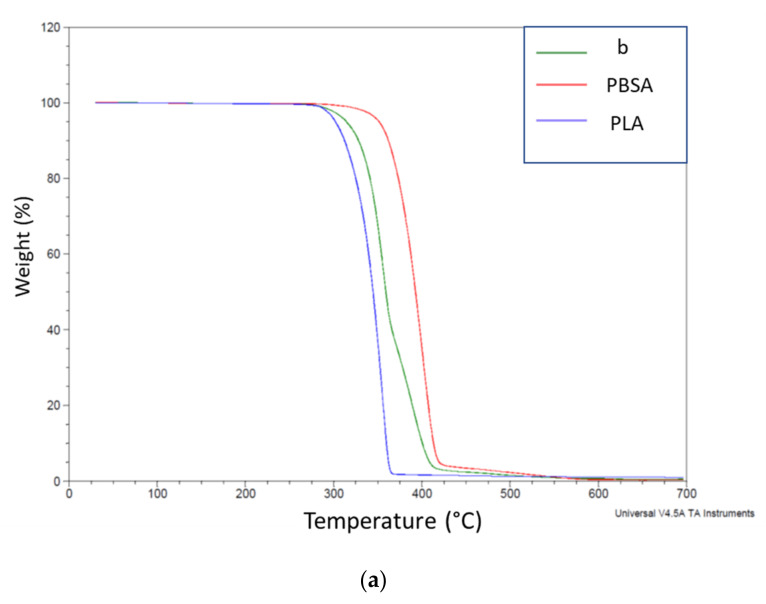

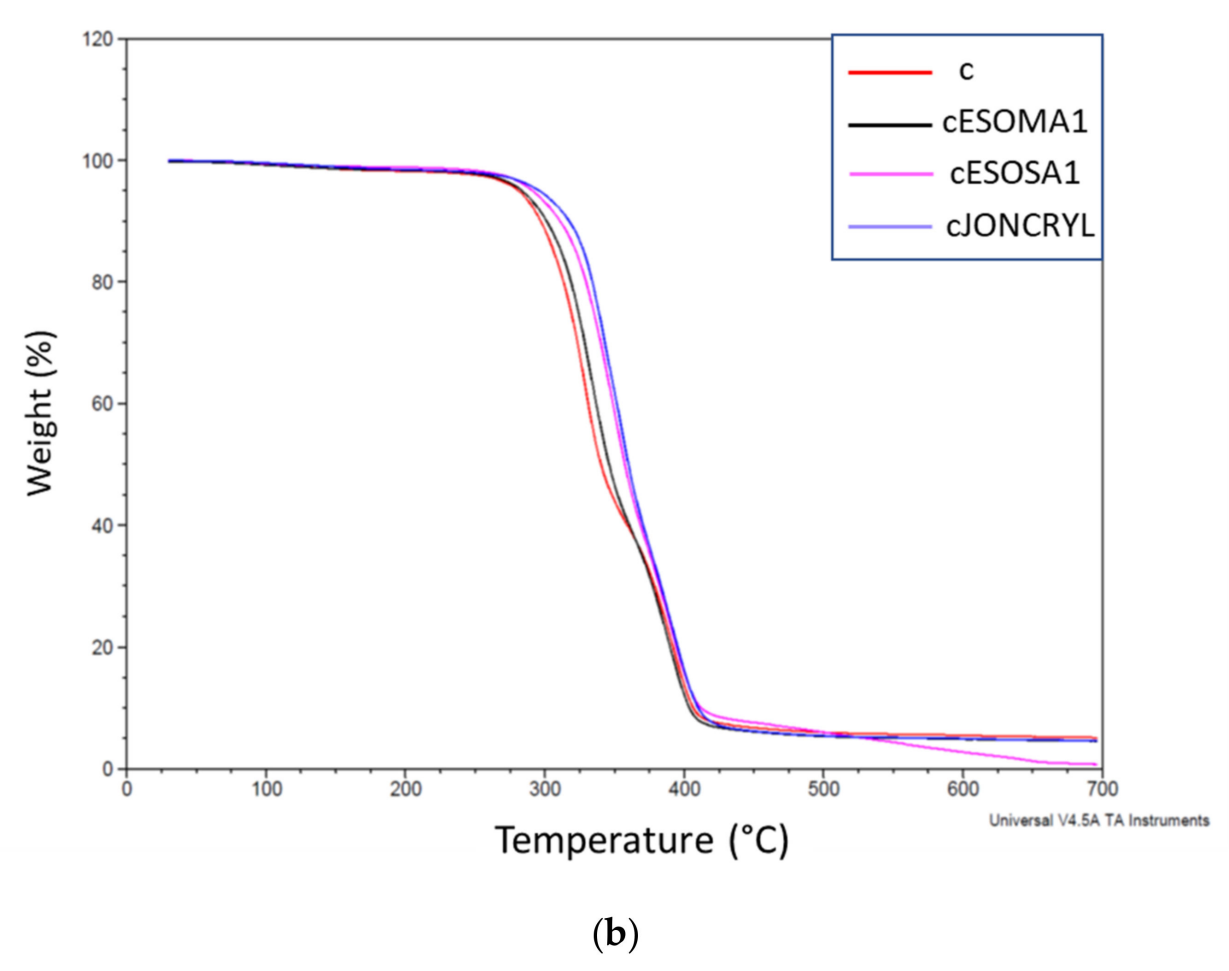
Thermograms of (**a**) pure polymers and (**b**) PLA/PBSA 60/40 blend.

Interestingly, two different thermal degradation steps could be identified in the thermograms of b blend (Figure 11a), attributable to PLA and PBSA, respectively. By elaborating the curves considering the derivative trend, it is possible to calculate the mass loss attributable to PLA and PBSA, respectively (Table 5). In the presence of ESO the peak temperature attributable to PLA is significantly reduced as well as the mass loss attributable to PBSA. When the chain extender systems were added, significant variations of peak temperature and PBSA mass loss could be evidenced, making the thermal behavior more similar to the unplasticized composite (c). These variations can be ascribed at the change in polymeric structure correlated at the chain extension systems reactivity. cESO and CESOSA1 showed a similar behavior, whereas cESOMA and cJONCRYL –developing a more branched structure– showed a similar increase in temperature of PLA degradation peak, similar to the not plasticized c composite. Thus, fully amorphous extensively branched systems, as resulted from DSC analysis, showed a similar behavior also in thermal degradation.

#### 3.3.4. Scanning Electron Microscopy (SEM) 

A morphologic investigation on cryo–fractured samples surfaces of the chosen formulations was carried out, to investigate changes of the phase distribution and of bran structure due to composition (Table 8).

Analyzing firstly b, the pure PLA/PBSA matrix, since the ratio was 60/40, it was evident that the two components were highly interpenetrated and the adhesion was quite high, in agreement with previous investigations [38]. Regarding c composite, wherein non–modified bran was added, at high magnification particles with different sizes and shapes and some agglomerates were identifiable and attributable to the characteristic structure of pure bran. In particular, some disc–like elements (reasonably starch granules [59]) were present, indicating a limited compatibility between the matrix and the fibers. Around some bran granules, demarcation, and crack lines can be found.

In cESO, the addition of ESO as unique modifier agent significantly enhanced the compatibility between fibers and matrix, due to its plasticizing effect and mild reactivity. Holes indicated the occurrence of the pull–out mechanism (mode of deformation which is related to fibers exit from the principal matrix, ensuring its mechanical resistance), typical of fiber–reinforced polymeric materials.

Looking at cESOMA1, SEM images, at low magnification it was quite complicated to obtain clear understanding of the interactions between fibers and matrix. This meant that the modification reaction exploiting malic acid worked, but at high magnification, even if in isolated points, big circular particles, reasonably starch granules, indicated that the curing process did not affect bran fibers in a uniform and complete manner. This kind of gaps indicate a low adhesion between the matrix and this starch components of bran.

Regarding cESOSA1 formulation (where succinic acid was employed), its distribution appeared much fine and bran fibers were difficult to be recognized because well incorporated in the matrix. Groups of small holes (like honeycombs) and the absence of single starch grains indicated that the covering of fibers by the SA–based modifier system was highly effective and extensive.

When Joncryl was exploited alone as fossil chain extender (cJONCRYL), the adhesion resulted the highest between the PLA and PBSA (as evident at high magnification), because Joncryl increases the phase compatibility in PLA/PBSA blends, but the surface modification of fibers was not improved with respect to cESOSA1, because starch molecules were again visible and cleavage lines could pass just through fibers themselves.

## 4. Discussion

**Figure 12:**
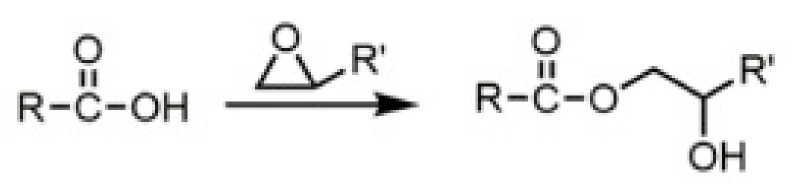
Ring opening reaction.

As already said, epoxidized vegetable oils, like ESO, can be cured with biobased dicarboxylic acids, to favor the formation of high molecular weight branched networks, completely biobased and biodegradable [40,46]. If the presence of ESO alone formulations performed mainly plasticization effects, the curing of this oil with diacids was performed according to the ratio between epoxy and –COOH groups, whose interaction led to the formation of bridges. In the reaction the epoxy ring opened hanging the carboxyl group to form an ester bond, following the pattern of a nucleophilic addition (Figure 12).

**Figure 13:**
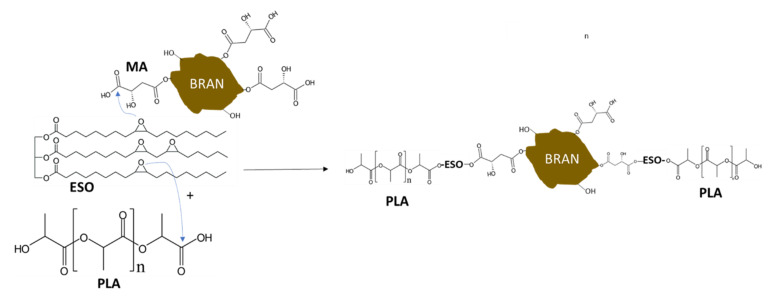
Reactions pattern between a bran particle, biopolyesters, MA, and ESO.

In practice, considering the polar malic acid, it performs physical interactions (for instance by hydrogen bonding) or grafting on bran, and its terminal carboxyl groups on the other side are ready to react with epoxy rings of ESO, which open out. On the other hand, ESO rings react in the same way with terminal carboxyl groups of PLA/PBSA blend. Rings opening leads to the formation of ester bonds and increases the number of ramifications, represented by skeletons of malic acid, ESO, and bran. The overall curing reaction can be thus considered an efficient chain extension, including reactive compatibilization. ESO and MA work as a unique compatibilizer system between fibers and the polymeric blend. This mechanism is confirmed by the higher effectiveness in melt fluidity decrease in bran composites than in blends (Figure 7 and Figure 8). The results showed that bran plays an important role in the branching mechanism. This mechanism is acceptable because reminds to an analogue process already defined in the case of PLA/maleic anhydride–grafted–starch blends, compatibilized by ESO. Starch is again a polysaccharide, rich of –OH groups (like bran filler), and maleic anhydride has a structure similar to malic acid one, but with a double bond [60]. A schematic pattern of the reaction is summarized in Figure 13.

An analogue reaction pattern was successively hypothesized when succinic acid was exploited as dicarboxylic acid in place of malic acid. The most significant difference is related to degradation effects caused by two acids. In fact, this kind of phenomena, which develop in competition with the desired chain extension reaction, is hindered in succinic acid–based formulations. In fact, being lack of the hydroxyl group, it does not favor early hydrolysis of polyester matrix as much as malic acid did. Hence, succinic acid better embodies requirements which had been described by Zeng and coworkers [46], improving properties of prepared blends and biocomposites. Looking at the comparison with MA and Joncryl, it can be considered a very promising alternative in chain extenders field, since it is fully biobased and cheap and it is able to ensure ideal values for melt flow parameters, similar to ones showed by pure matrix of PLA/PBSA (b formulation).

Interestingly in ESO +MA system the stoichiometric ratio is the most advantageous for having a good chain extension. On the other hand, in both ESO + MA and ESO + SA systems the best results in terms of melt fluidity reduction are achieved by adding 0.72–0.73% of reagents in stoichiometric ratio. This represents a limitation in this bran composites and makes the melt fluidity not fully modulable. The reason why this minimum exists is difficult to explain, but it is reasonably linked at hydrolysis kinesis, heavily influenced by the DCA content in the system. On the other hand, bran represents a filler that tends to easily promote biopolyesters chain scission because of its complex composition, including proteins and starch. Thus, the further addition of acids, yet indicated as reagents inducing hydrolysis in PLA [61,62] is particularly disadvantageous.

As observed from TGA thermograms, it is possible to conclude that the degradation of biocomposites depends on the thermal stability of the secondary additives (dicarboxylic acids), which showed onset temperatures even lower than extrusion temperature and catalyzed polyester matrices decomposition already during processing. Since values of Ton were higher than 240 °C for all blends, it would be logical to set at about 230–240 °C the limit temperature of practical use. This is in agreement with the literature, from which it is known that processes and applications of natural fibers composites should be restricted at a maximum of 250 °C [63]. Moreover, it is clear to observe that the incorporation of hydrophilic bran fibers (even modified) led to a decrease (even if slight) in the thermal stability of the original matrix, but for this reason it could be advantageous for improving thermal decomposition properties of composites as well [64].

From DSC studies, not significant changes of pure PLA/PBSA matrix thermal characteristics were observed when bran was added and chain extender systems were employed. The crystallization was, on the whole, slightly promoted using ESO + SA but the chain extended final materials resulted mainly amorphous.

## 5. Conclusions

Biobased chain extenders were formulated by combining epoxidized soybean oil (ESO) and dicarboxylic acids (DCA), in particular malic acid (MA) and succinic acid (SA).

Thanks to thermogravimetric and spectroscopic studies it was possible to verify the reactivity between ESO and the acids at the temperature typical of polyester composites processing. Then, thanks to miniextruder torque and melt fluidity analysis, it was possible to verify the occurrence of the reaction in the melt during the processing of both PLA/PBSA 60/40 blend and its composite including 20 wt % of wheat bran.

As bran much affects the processability of biopolyester blends, because of induced chain scissions, the biobased chain extenders were validated in a severe system. The obtained results indicated that linking the new chain extension systems on fillers, following the stoichiometric ratio between epoxide and carboxylic groups in ESO and DCA, allowed to achieve better results. At this purpose, a minimum point for melt fluidity at 0.7–0.8% by weight (hence close to 1%) of chain extension system was found for both ESO + MA and ESO + SA in these bran composites. Despite this represents a limit in the tailoring of melt fluidity, by using ESO and SA values allowing biopolyesters processing and an improved melt stability were achieved.

The mechanical properties are not much significantly affected by the different chain extension systems, despite good elongation at break values were reached adding ESO + SA. Regarding thermal properties, the slight nucleating effect of bran is reduced by ESO + MA and slightly improved by ESO + SA, suggesting a parallelism with the occurrence of more extensive disordering by branching using MA, due to its –OH group.

The commercial Joncryl resulted more efficient in terms of melt fluidity reduction, but it is necessary to consider that Joncryl oligomers contain about 20 epoxydic groups for each molecule, whereas the ESO has 4 epoxide groups for each molecule on average. The capacity of Joncryl of chain extending and branching polymers is thus necessarily higher. Despite this difference, thanks to the cross–linking action on ESO played by the SA, the ESO/SA system assumes a behavior similar to the one of Joncryl considering the difference in epoxydic groups number. The average number of reactive groups per chain extender molecule is increased thanks to the reaction between epoxidized oils and dicarboxylic acids. 

These biobased chain extenders are promising for the processing of biobased composites and they could be also considered in the future to allow their recycling. In fact, using not bio–based chain extenders can be detrimental for the biodegradability of the material. Thanks to this approach composites could be processed and then, in their final stage of life, they can be fully composted overcoming the issue of possible micro–plastic residues.

## Figures and Tables

**Table 1:** Weight compositions of prepared blends.

Sample	PLA/PBSA (wt %)	Bran (wt %)	ESO (wt %)	MA (wt %)	SA (wt %)	Joncryl (wt %)	Extender Total Weight %
b	100	–	–	–	–	–	–
c	80	20	–	–	–	–	–
cESO	80	19	1	–	–	–	–
cESOMA1	80	19	0.78	0.22	–	–	1
cESO > MA1	80	19	0.88	0.12	–	–	1
cESOMA5	80	15	3.9	1.1	–	–	5
bESOMA1	99	–	0.78	0.22	–	–	1
cESO < MA1	80	19	0.68	0.32	–	–	1
cESOMA2	80	18	1.56	0.44	–	–	2
cESOMA05	80	19.5	0.39	0.11	–	–	0.5
cESOSA05	80	19.5	0.4	–	0.1	–	0.5
cESOSA1	80	19	0.8	–	0.2	–	1
cESOSA2	80	18	1.6	–	0.4	–	2
cESOSA5	80	15	4	–	1	–	5
bESOSA1	99	–	0.8	–	0.2	–	1
cJONCRYL	80	19	–	–	–	1	1

**Table 2:** Thermogravimetry data regarding reagents, bran and modified bran.

Sample	Wt. Loss (%)	Residue (%)	T Onset (°C)	Tpeak (°C)
ESO	96.9	2.8	262.2	371.9
MA	98.3	1.2	131.4	231.2
SA	97	2.9	148	215.6
Bran	9.8; 70.1	19.9	170.2	307.4
ESO/MA bran	7.1;72.2	19.5	162.9	302.2
ESO/SA bran	6.2; 67.8	24.7	172.8	302.2

**Table 3:** Wavenumbers related to the main infrared ATR peaks of MA, SA, and ESO.

Vibration	MA (cm^−1^)	SA (cm^−1^)	ESO (cm^−1^)
C–H stretching	2873	–	2924; 2855
C=O stretching	1687	1669	1741
Stretching of –C–C=O bond	1115 (double)	1195	1151
Third isolated –C–O(H) stretching	1095	–	–
Out–of–plane bending of –COOH	930	910	–
Epoxy rings	–	–	834; 826

The sign–indicates not detectable peaks.

**Table 4:** Torque and melt flow values of prepared blends and biocomposites.

Sample	Torque (N·cm)	MFR (g/10 min)	MVR (cm^3^/10 min)	Melt Density (g/cm^3^)
b	99.5 ± 0.8	5.5 ± 0.4	5.2 ± 0.4	1.058
c	84.7 ± 0.8	10.3 ± 1.5	10.8 ± 1.6	0.956
cESO	79.5 ± 1.7	10.1 ± 1.3	10.3 ± 1.3	0.973
cESOMA1	94.5 ± 1.4	8.0 ± 1.0	8.3 ± 1.1	0.955
cESO > MA1	93.9 ± 0.7	8.8 ± 0.8	8.5 ± 0.8	1.034
cESOMA5	61.0 ± 1.0	24.2 ± 2.4	24.9 ± 2.4	0.973
bESOMA1	74.7 ± 1.2	14.0 ± 1.2	14.2 ± 1.2	0.991
cESO < MA1	80.2 ± 0.8	9.9 ± 1.3	10.2 ± 1.3	0.976
cESOMA2	82.0 ± 1.2	10.1 ± 0.03	10.3 ± 0.3	0.978
cESOMA05	91.0 ± 1.2	8.4 ± 0.2	8.7 ± 1.1	0.967
cESOSA05	101.7 ± 1.5	5.2 ± 0.01	5.2 ± 0.5	0.981
cESOSA1	99.0 ± 1.4	5.4 ± 0.1	5.5 ± 0.7	0.982
cESOSA2	91.5 ± 1.3	6.1 ± 0.03	6.5 ± 1.1	0.938
cESOSA5	59.7 ± 2.1	10.0 ± 0.5	11.8 ± 2.1	0.847
bESOSA1	104.0 ± 1.6	3.5 ± 0.1	3.7 ± 0.7	0.946
cJONCRYL	114.5 ± 1.0	2.2 ± 0.3	2.3 ± 0.2	0.956

**Table 5:** Mechanical parameters of the selected formulations.

Sample	σ_y_ (MPa)	σ_b_ (MPa)	ε_b_ (%)
b	35.8 ± 1.6	24.5 ± 1.3	31.9 ± 1.6
c	28.8 ± 0.7	23.9 ± 0.8	12.0 ± 1.0
cESO	26.7 ± 1.2	21.8 ± 1.2	20.9 ± 2.9
cESOMA1	28.1 ± 1.0	23.2 ± 0.9	16.3 ± 1.2
cESOSA1	24.5 ± 0.8	20.1 ± 0.8	23.1 ± 1.9
cJONCRYL	33.5 ± 1.1	28.3 ± 0.7	18.4 ± 1.6

**Table 6:** Thermal features derived from DSC analysis of b, c, cESO, cESOMA1, cESOSA1, and cJONCRYL.

Sample	PLA Tg (°C)	PBSA Tm (°C)	PBSA ΔHm (J/g)	PLA Tcc (°C)	PLA ΔHcc (J/g)	PLA Tm (°C)	PLA ΔHm (J/g)	Xc (%)
b	58.4	87.4	14.6	117.7	16.2	151.6	16.7	0.9
c	56.1	87.1	14.1	107.5	14.7	148.6	17.1	6.9
cESO	55.8	86.9	12.7	117.3	13.5	151.1	15.4	4.2
cESOMA1	55.9	86.7	12.5	117.1	14.6	151.2	15.1	1.1
cESOSA1	56.5	86.1	12.4	111.9	13.4	149.5	16.2	6.2
cJONCRYL	57.9	86.3	10.4	118.7	11.4	151.1	11.6	0.4

**Table 7:** TGA results for pure PLA and PBSA and for F1, F2, F3, F4, F12, and F16 formulations.

Sample	Tonset (°C)	Tpeak (°C)	Total Loss (wt %)	PLA Loss (wt %)	PBSA Loss (wt %)	Residue (wt %)
PLA	274.1	354.5	97.58	97.6	–	2.4
PBSA	301.3	401.1	95.72	–	95.7	4.3
b	261.3	355.4; 391.2	97	64.7	32.3	2.7
c	242.9	355.4; 388.8	90.6	57.8	32.8	7.2
cESO	247.6	328.6; 391.7	90.7	62.5	28.2	7.4
cESOMA	243.4	350.2; 389.3	91.5	58.4	33.1	6.4
cESOSA	244.3	333.8; 389.3	90.5	61.5	29	7.9
cJONCRYL	253	349.8; 390.7	91.8	61.2	30.6	6.1

**Table 8:** Comparison between SEM images of b, c, cESO, cESOMA1, cESOSA1, and cJONCRYL formulations: low magnification factor (left) versus high magnification factor (right).

Sample	Low Magnification	High Magnification
b	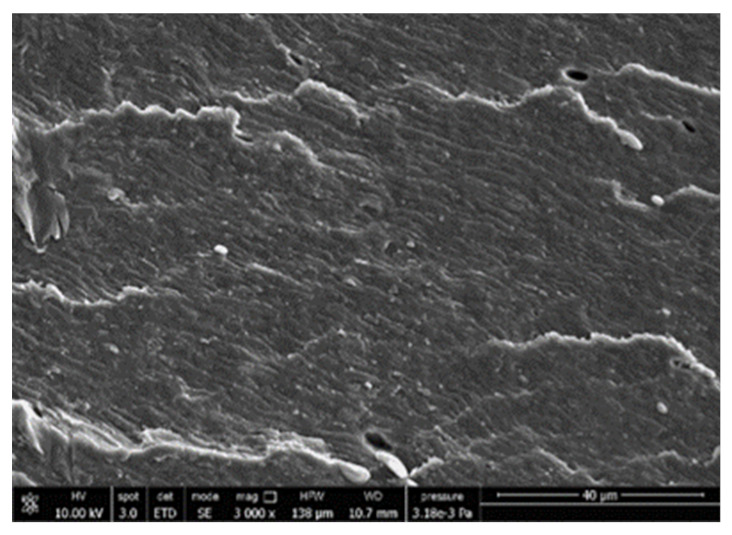	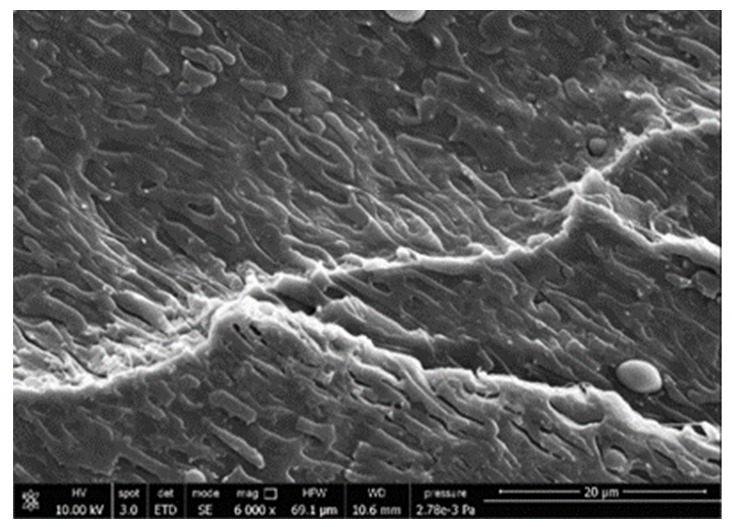
c	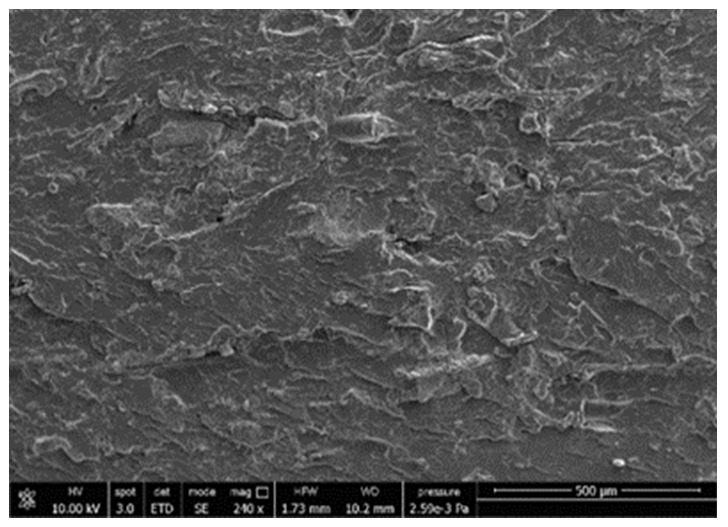	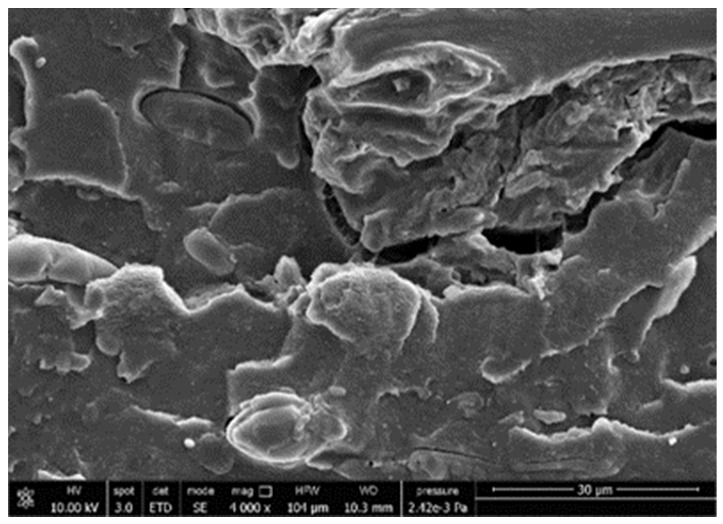
cESO	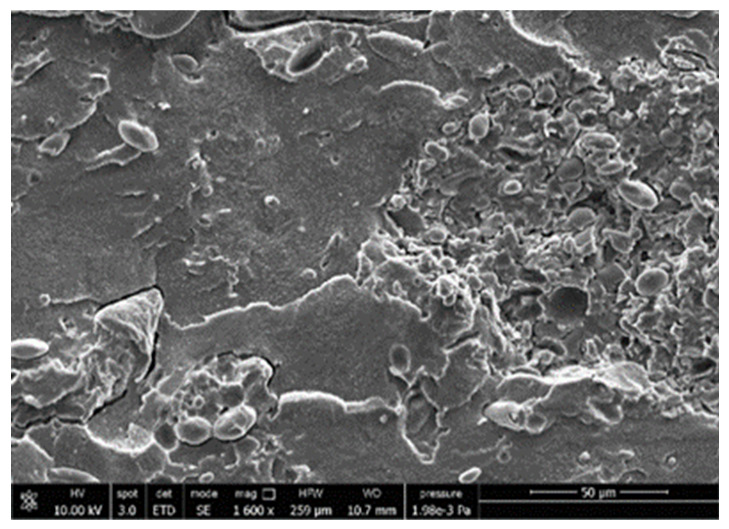	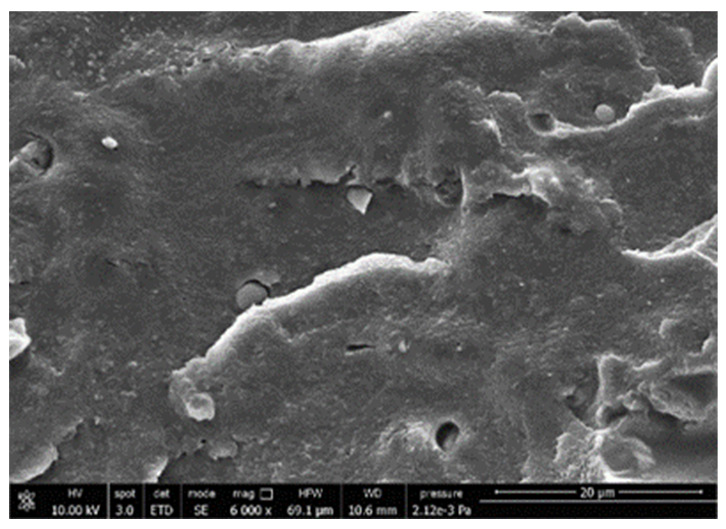
cESOMA1	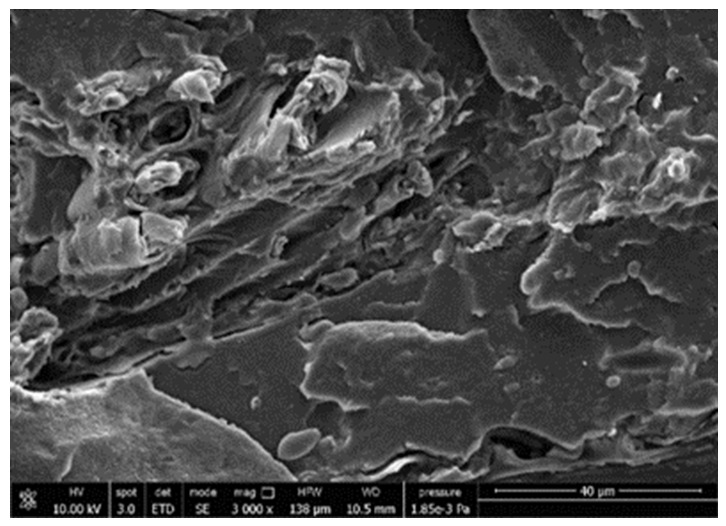	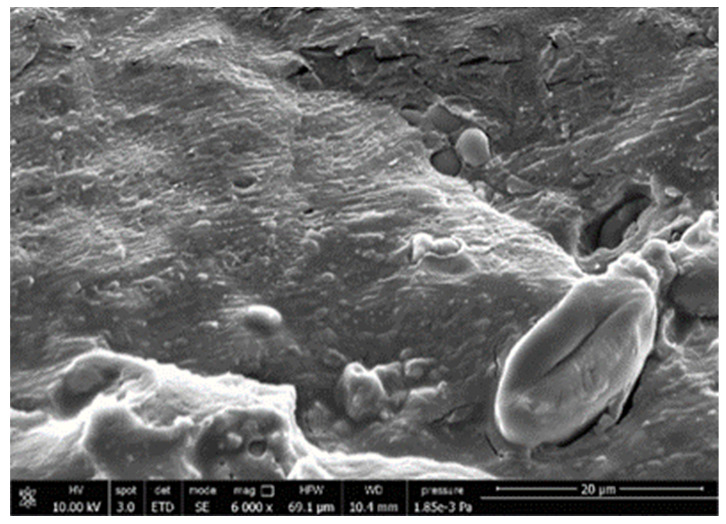
cESOSA1	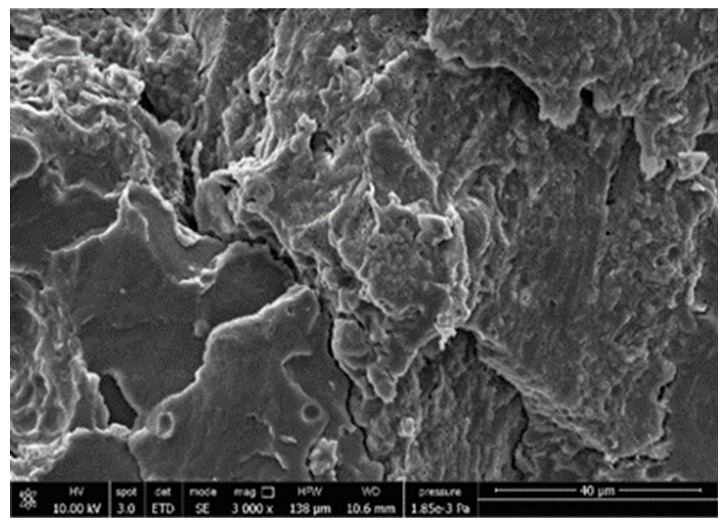	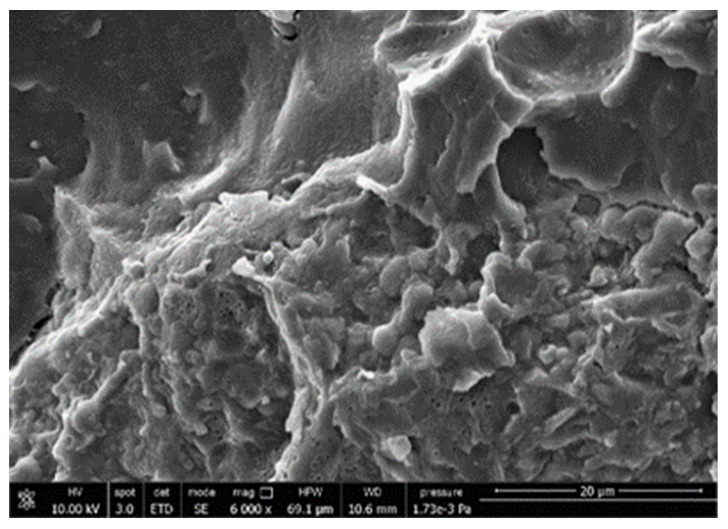
cJONCRYL	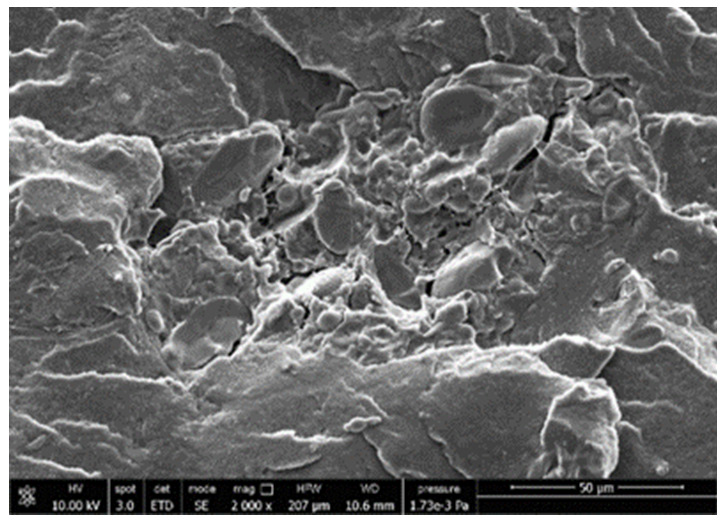	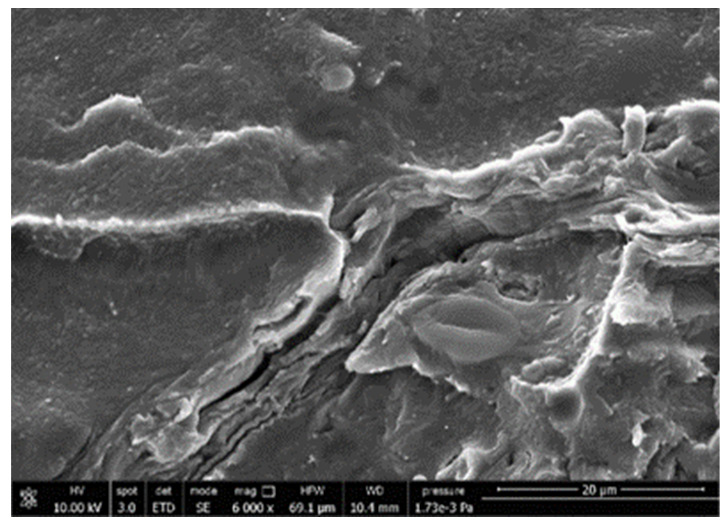

## Data Availability

The data presented in this study are available on request from the corresponding author.
